# Local Government Competition and Regional Green Development in China: The Mediating Role of Environmental Regulation

**DOI:** 10.3390/ijerph17103485

**Published:** 2020-05-16

**Authors:** Na Zhang, Jinqian Deng, Fayyaz Ahmad, Muhammad Umar Draz

**Affiliations:** 1 School of Economics, Lanzhou University, Lanzhou 730000, China; zhangn19@lzu.edu.cn (N.Z.); fayyaz@lzu.edu.cn (F.A.); 2 Independent Researcher, Scarborough, ON M1L 4P2, Canada; umardraz2626@gmail.com

**Keywords:** local government competition, green development efficiency, environmental regulation, China, SBM-DEA

## Abstract

Green development is an important way to meet the challenges of ecological and environmental protection and economic growth, as well as an inevitable choice to realize China’s sustainable development in the new era. The Chinese economic system is such that local government competition has become a key factor affecting regional green development under the current leadership. Based on the inter-provincial panel data of 30 provinces in mainland China from 1997 to 2017, this paper uses the total-factor non-radial directional distance function and slack-based measure data envelopment analysis (SBM-DEA) to measure the green development efficiency of the provinces. Additionally, it also uses the Malmquist–Luenberger (ML) index to decompose green development efficiency and analyzes its internal driving factors. Finally, taking environmental regulation as a mediating variable, this paper empirically analyzes the influence mechanism of local government competition on green development efficiency from three perspectives including growth competition, fiscal competition and investment competition. The study found that: the green development efficiency of Chinese regions showed a downward trend, with significant regional differences; technological progress is the key factor to improve the efficiency of green development, and its role gradually decreases from eastern to western and central regions; pure technical efficiency has become a bottleneck restricting the improvement of green development efficiency, while scale efficiency shows significant regional differences; the growth competition, fiscal competition and investment competition of local government all have a significant inhibitory effect on the efficiency of green development. This paper puts forward policy suggestions supporting enterprise technology research and development, optimizing energy conservation and emission reduction as well as improving the local government performance evaluation system for green development.

## 1. Introduction

**Figure 1:**
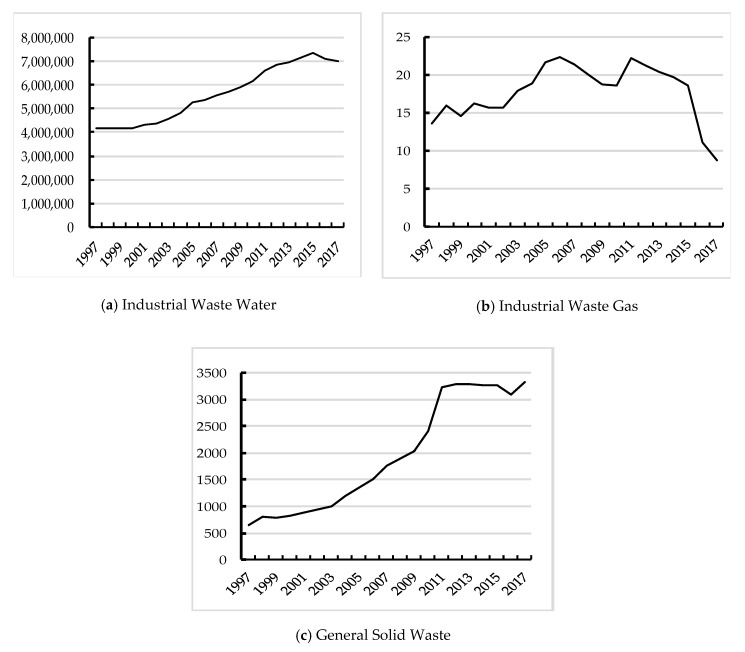
Change trend of the three industrial wastes in 1997–2017. (y-axis unit: million tons) (Since the emission of industrial waste gas is only counted to 2010 in the China Statistical Yearbook, the emission of main pollutants in the waste gas starts in 2011. In order to ensure the consistency of data, the emission of industrial sulfur dioxide is used as the alternative indicator for the emission of industrial waste gas.)

Chinese urbanization and industrialization have experienced 40 years of reforms and opening up, and the level of urbanization reached 59.58% in 2018. According to the Northam curve, China’s urbanization has entered an accelerated stage. However, with the rapid agglomeration of urban population and the continuous expansion of urban space, urban development has deviated from the sustainable development track of high quality and high efficiency. With the development of industrialization, the Chinese economy has achieved a miracle in the history of world economic growth with “factor input driving” [1], and has leaped to being the second largest economy in the world with an average growth rate of nearly 10% per annum. However, the extensive growth mode of high investment, high energy consumption and high pollution mean China faces severe problems such as tight resource constraints, serious environmental pollution, and ecosystem degradation. According to the data of the bulletin of China’s ecological environment in 2018, only 35.8% of 338 prefecture-level cities met air-quality standards, resulting in 1899 days of severe air pollution and 822 days of severe pollution; only 13.8% of the groundwater has reached the three standards in the groundwater quality test, and some basins such as the Yellow River still suffer light or moderate pollution. According to the data of industrial waste water, waste gas and solid waste in the China Statistical Yearbook, from 1997 to 2017, the discharge of industrial waste water and the production of general solid waste decreased in some years, but the overall trend was rising. Among them, the discharge of industrial waste water increased from 41,580.7 million tons in 1997 to 69,966.1 million tons in 2017, and reached the highest level in 2015, with 73,500.0 million tons. The production of general solid waste increased from 657.5 million tons in 1997 to 3315.92 million tons in 2017. However, the emission of industrial waste gas showed a fluctuating downward trend, from 13.63 million tons in 1997 to 8.75 million tons in 2017. As shown in Figure 1. The pollution problem in China is particularly serious, which not only causes serious harm to public health, but also seriously affects the international image of the Chinese government.

Industrialization has created unprecedented material wealth, but also produced irreparable ecological trauma” [2]; as the return on investment of traditional elements continues to decline, China’s economic growth urgently needs to transform to green development based on technological innovation. Since 2012, China’s economic growth has begun to enter a new normal, and the pressure of economic transformation is imminent. The Fifth Plenary Session of the 18th Communist Party of China National Congress proposed the new development concept of “innovation, coordination, green, opening and sharing”. At present, green development has become the main direction of China’s economic transformation. The essence of green development is to regard resources and environment as the endogenous factors of growth, and provide the balance between economic growth and ecological environment protection by changing the dynamic mechanism of economic development, so as to form a new sustainable development model [3,4]. By contrast with the western governance system, under the mechanism of fiscal decentralization in China local government has become the main body of regional green development, and the choices of local government have greatly affected the efficiency of regional green development. In addition, the Chinese governance model of “political centralization + economic decentralization” makes local governments compete in economic, financial, investment and other aspects. Therefore, the core focus of this paper is to seek answers for the following questions: how does local government competition affect the efficiency of regional green development; and can we achieve a win-win situation between economic growth and environmental protection? This study is expected to provide empirical evidence and a decision-making reference for regulating the behavioral choices of local governments and promoting the future regional green development in China.

The paper unfolds as follows: Section 2 presents a literature review. Section 3 is based on the slack-based measure data envelopment analysis (SBM-DEA) model to measure the efficiency of China’s regional green development, and is based on the Malmquist-Luenberger (ML) index analysis to discuss the driving factors of green development. Section 4 describes the variables and data processing, then based on the research problems constructs the mediating effect model. Section 5 shows empirical results about the impact of local government competition on the efficiency of regional green development with environmental regulation as mediating variable. Section 6 summarizes the whole paper and discusses the policy implications of its conclusions.

## 2. Literature Review

With the development of industrial civilization and the accumulation of material capital, environmental issues have increasingly attracted the attention of countries around the world, and the green development mode of resource saving and environment friendly activity has become the focus of economic development in various countries and regions [5]. To improve the efficiency of green development has become an inevitable choice for China to overcome the multiple dilemmas of resources, environment and economic development. In recent years, many scholars have studied the green development mode and its influencing factors, and the review of the literature will be classified into different sections.

### 2.1. The Conceptual Evolution of Green Development

The concept of green development can be traced back to the spaceship economic theory put forward by Boulding in 1966 [6]. He believed that the “circular economy” must be established to replace the traditional “single program economy”. In 1989, Pearce et al. [7] put forward the concept of “green economy”, which is defined as “an economic development mode that can be sustained by the natural environment and human beings, and that does not lead to ecological crisis and social division due to human’s blind pursuit of economic growth, and does not lead to the unsustainable development of economy due to the depletion of natural resources”. Since then, many scholars have carried out a lot of research on energy efficiency and environmental pollution in economic development [8,9]. Pierre-André and Perthuis [10] believe that green development will change the mode of production and consumption, maintain and restore the Earth’s natural resources, and regard the environment as an essential element of production rather than an exogenous objective existence. Sabit [11] pointed out that the transformation from economic growth to green development mode is a major challenge facing the world today. The research report on China’s green development in the 21st century explains the concept of green development in China, that is, on the premise of the sustainability of natural capital, we should try our best to replace environmental capital and natural capital with man-made capital, constantly improve the utilization efficiency of environmental resources, and realize the development mode of low material consumption and low energy consumption. Therefore, for China, a developing country with a dense population, shortage of resources and serious environmental pollution, green development aims to improve the utilization efficiency of capital, labor, energy and other production factors while achieving economic growth through the application of green technology, and reduce the emissions of pollutants such as waste water, waste gas and solid waste [12]. This is also the green development mode advocated by the Chinese government at present.

### 2.2. Measurement of Green Development and Its Influencing Factors

In recent years, research on green development has become a hot spot, mainly including, firstly, the calculation of green development efficiency. The existing literature mainly adopts two methods to measure green development: the efficiency method and index method. (1) The efficiency method uses data envelopment analysis (DEA), and based on the framework of total factor production, emphasizes to obtain as much economic output as possible at the cost of environmental resources as little as possible [13,14]. Among them, part of the study adopted the input-output index system developed by Word Business Council for Sustainable Development (WBCSD), which takes the unexpected output of environmental pollution as the input variable into the model, and obviously deviates from the actual situation [15]. In order to make the estimation more accurate, many scholars have improved the DEA model. Zhou [16] proposed the non-radial direction distance function (NDDF) based on the direction distance function (DDF), and then Zhang et al. [17] put capital and labor into the environmental efficiency analysis, and proposed the total-factor non-radial directional distance function (TNDDF). Tone [18] proposed the slack-based measure (SBM) model including unexpected output, which can evaluate the efficiency including multiple inputs, expected output and unexpected output. (2) The index method calculates the efficiency of green development by constructing a multiple index system. Li et al. [19] used the human development index for reference, and calculated and ranked the green development index of 123 countries from the two dimensions of economic society and sustainable development of resources and environment. Sun et al. [20], based on the information entropy model and the theory of dissipative structure, established an evaluation index system to calculate the green development level. Secondly, research has explored the influencing factors of green development. Environmental regulation has become the focus of discussion. Based on the data of American manufacturing industry, Brunermeier and Cohen [21] use pollution reduction expenditure and government supervision and law-enforcement activities to measure the environmental regulation, and find that pollution reduction expenditure significantly promotes green development, but government supervision and law-enforcement activities cannot effectively stimulate green development. Horbach [22] confirms that environmental regulation is an important driving force of green innovation based on German data. Langpap and Shimshack [23] and Cole et al. [24] both believe that informal environmental regulations such as public supervision play a significant role in green development. In addition, some scholars discussed the influence of urbanization [25], finance [26], human capital [27] and industrial structure [28] on green development.

### 2.3. Local Government Competition and Green Development

Many studies have shown that local government competition, characterized by “promotion tournament”, is an important political foundation to promote China’s rapid economic growth in the socialist market economy with Chinese characteristics [29,30]. Firstly, in the context of fiscal decentralization, local governments must adopt the strategy of “opening up resources and reducing expenditure” to relieve financial pressure. On the open source side, local governments reduce environmental regulation standards to attract investment from enterprises [31], thus increasing fiscal revenue. In terms of reducing expenditure, local governments have repeatedly reduced the financial expenditure on non-economic public goods such as environmental governance. Therefore, some studies show that the competition of local governments caused by fiscal decentralization is not conducive to the green development of the regional economy [32,33]. Secondly, local government competition is likely to cause the “bottom-by-bottom effect” of environmental regulation between regions. On the one hand, environmental regulation will increase the burden of enterprises, thus producing a crowding out effect on local enterprises, which is not conducive to regional economic growth [34]. On the other hand, environmental regulations will improve the efficiency of resource utilization and reduce pollution emissions, which will have a positive impact on regional green development [35]. However, some scholars argue that devolution has not led to a bottom-line of competition between governments [36,37]. Finally, local government competition is likely to lead to the prevalence of local protectionism, enhances the externality of environmental pollution, and makes it difficult for regional collaborative governance. Therefore, Stewart [38] believes that environmental regulation must rely on the central government to avoid the “tragedy of the commons”, and that the environmental protection agency has the power to veto unreasonable licensing decisions of the state in accordance with environmental law [39].

In summary, although some studies have measured the efficiency of green development in China, their time span is short and lack analysis of its driving factors. In addition, the existing research mainly discuss the influencing factors of green development from the aspects of environmental regulation and pollution reduction. Few studies put local government competition and green development into a systematic analysis framework, and there is no literature that comprehensively considers the multi-dimensional characteristics of local government competition in China to analyze the mechanism of local government competition affecting green development. Therefore, this paper chooses “green development efficiency” to measure the level of green development, which not only inspects economic development but also considers resource conservation and ecological protection, analyzes the current situation of regional green development in China, and explores the driving factors of green development based on ML index decomposition. On this basis, the paper examines the impact of local government competition on the efficiency of regional green development from three dimensions: growth competition, fiscal competition and investment competition, in order to provide experience for improving local government assessment mechanism and promoting green development.

The marginal contribution of this paper is as follows: (1) this paper measures the efficiency of green development in China since 1997, and further decomposes it into technological progress, pure technological efficiency and scale efficiency, which is helpful for a more comprehensive understanding of China’s long-term green development level, regional differences and the driving factors behind them, and provides reliable empirical evidence for further promoting green development. (2) From a new perspective, this paper studies the effect of local government behavior on the efficiency of green development under the Chinese government-dominated system. As the main body of green development in China, local governments compete in economic growth, finance, investment and other aspects of their jurisdiction, which greatly affects the efficiency of green development. Research on the influence of local government competition on the efficiency of green development broadens the research perspective of green development. (3) By introducing environmental regulation into the empirical model and analyzing its mediating effect between local government competition and green development efficiency, we can understand the mechanism of local government competition affecting green development efficiency more accurately.

## 3. Measurement and Decomposition of Green Development

Green development is the core issue of this paper. First, we use the total-factor non-radial directional distance function and SBM-DEA model to measure the green development efficiency of the province in China, and then use the Malmquist–Luenberger index to decompose the green development efficiency and analyzes its internal driving factors.

### 3.1. Methods

#### 3.1.1. Total-Factor Non-Radial Directional Distance Function and Slack-Based Measure Data Envelopment Analysis (SBM-DEA) Model

The improvement of green development level means the enhancement of green development efficiency, which can be achieved in two ways: (1) under the same input factors, the expected output increases while the unexpected output decreases; (2) under the premise that the input factors remain unchanged, the expected output remains unchanged while the unexpected output decreases [9]. For China’s green development, it is to reduce the emissions of waste water, waste gas and solid wastes while achieving economic growth on the premise that capital (k), labor (l) and energy (e) factor inputs remain unchanged. Therefore, we suppose there are N decision-making units, with k, l, e as the input elements, economic growth as the expected output (y), and pollutant emissions as the unexpected output (b). A production technology is defined as:

(1) $$T=\left\{(k,l,e,y,b):\sum_{n=1}^{N}z_{n}k_{n}\leq k,\sum_{n=1}^{N}z_{n}l_{n}\leq l,\sum_{n=1}^{N}z_{n}e_{n}\leq e,\sum_{n=1}^{N}z_{n}y_{n}\geq y,\sum_{n=1}^{N}z_{n}b_{n}=b,\right\}$$

where $z_{n}$ is the strength variable that links the input and output vectors to form a convex set, $z_{n}\geq 0,n=1,2,3,\cdots ,N$.

Further, the non-radial directional distance function is defined as:

(2) $$\vec{D}(k,l,e,y,b;g)=\sup\{w^{t}a:(y+\alpha_{y}g_{y},b-\alpha_{b}g_{b})\in p(k-\alpha_{k}g_{k},l-\alpha_{l}g_{l},e-\alpha_{e}g_{e})\}$$

Among them, $g=(-g_{k},-g_{l},-g_{e},g_{y},-g_{b})$ is the direction vector, $w^{t}=(w_{k},w_{l},w_{e},w_{y},w_{b})$ is the index weight, $a=(\alpha_{k},\alpha_{l},\alpha_{e},\alpha_{y},\alpha_{b})$ is relaxation vector, satisfying a≥0. Equation (2) can be interpreted as: when the production technology is unchanged, the producer wants to reduce the factor input along the direction of $-g_{k}$, $-g_{l}$, $-g_{e}$, increase the expected output along the direction of $g_{y}$, and reduce the unexpected output along the direction of $-g_{b}$. Then, the following DEA model can be built:

(3) $$\begin{array}{l} \vec{D}(k,l,e,y,b;g)=\max.w_{k}\alpha_{k}+w_{l}\alpha_{l}+w_{e}\alpha_{e}+w_{y}\alpha_{y}+w_{b}\alpha_{b}\\ s.t.\sum_{n=1}^{N}z_{n}k_{n}\leq k-\alpha_{k}g_{k},\sum_{n=1}^{N}z_{n}l_{n}\leq l-\alpha_{l}g_{l},\sum_{n=1}^{N}z_{n}e_{n}\leq e-\alpha_{e}g_{e},\\ \sum_{n=1}^{N}z_{n}y_{n}\geq y+\alpha_{y}g_{y},\sum_{n=1}^{N}z_{n}b_{n}=b-\alpha_{b}g_{b} \end{array}$$

When $\vec{D}(k,l,e,y,b)=0$, it means that the decision-making unit is located on the frontier of production just along the direction $g=(-g_{k},-g_{l},-g_{e},g_{y},-g_{b})$. Assuming that input factors, expected output and unexpected output are equally important [40], then the weight matrix of all indicators is $w^{t}=(\frac{1}{9},\frac{1}{9},\frac{1}{9},\frac{1}{3},\frac{1}{3})$, and substituting into Equation (3), the optimal relaxation variable of the n-th decision-making unit is $a_{n}^{*}=(\alpha_{nk}^{*},\alpha_{nl}^{*},\alpha_{ne}^{*},\alpha_{ny}^{*},\alpha_{nb}^{*})$, and further calculating the total-factor efficiency index of the n-th decision-making unit as follows:

(4) UEIn=14[yn/kn(yn+αny*yn)/(kn−αnk*kn)+yn/ln(yn+αny*yn)/(ln−αnl*ln)+yn/en(yn+αny*yn)/(en−αne*en)+yn/bn(yn+αny*yn)/(bn−αnb*bn)]=14[(1−αnk*)+(1−αnl*)+(1−αne*)+(1−αnb*)1+αny*]=1−14(αnk*+αnl*+αne*+αnb*)1+αny*,n=1,2,⋯,N.

Among them, $UEI_{n}\in [0,1]$, the closer to 1, the higher the energy and environmental efficiency of the n-th decision-making unit, when $UEI_{n}=1$, it shows that the n-th decision-making unit is on the frontier of green production, and the factor input efficiency reaches the highest level.

#### 3.1.2. Malmquist–Luenberger Index

Based on the directional distance function, according to the ML index proposed by Chung et al. [41], the ML productivity index of t+1 period based on t can be expressed as:

(5) $$ML_{t}^{t+1}={\left\{\frac{1+\vec{D_{0}^{t}}(k^{t},l^{t},e^{t},y^{t},b^{t};g^{t})}{1+\vec{D_{0}^{t}}(k^{t+1},l^{t+1},e^{t+1},y^{t+1},b^{t+1};g^{t+1})}\times \frac{1+\vec{D_{0}^{t+1}}(k^{t},l^{t},e^{t},y^{t},b^{t};g^{t})}{1+\vec{D_{0}^{t+1}}(k^{t+1},l^{t+1},e^{t+1},y^{t+1},b^{t+1};g^{t+1})}\right\}}^{\frac{1}{2}}$$

Among them, $ML_{t}^{t+1}>1$ represents a growth in green total factor productivity (TFP), and $ML_{t}^{t+1}<1$ represents a decrease in green TFP. $\vec{D_{0}^{t}}(k^{t},l^{t},e^{t},y^{t},b^{t};g^{t})$ and $\vec{D_{0}^{t+1}}(k^{t+1},l^{t+1},e^{t+1},y^{t+1},b^{t+1};g^{t+1})$ represent the distance function of t and t+1, $\vec{D_{0}^{t}}(k^{t+1},l^{t+1},e^{t+1},y^{t+1},b^{t+1};g^{t+1})$ is the mixed distance function of t+1 period based on the technology of t period, $\vec{D_{0}^{t+1}}(k^{t},l^{t},e^{t},y^{t},b^{t};g^{t})$ is the mixed distance function of t period based on the technology of t+1 period.

Furthermore, the change of the ratio of efficiency to productivity of the decision-making unit can be decomposed into the change of technological progress ($TC_{t}^{t+1}$) and the change of technological efficiency ($TEC_{t}^{t+1}$), then Equation (5) can be expressed as: $ML_{t}^{t+1}=TC_{t}^{t+1}\times TEC_{t}^{t+1}$. We relax the constraint of constant return to scale (CRS), then under the condition of variable return to scale (VRS), the change of technical efficiency can be further decomposed into two parts: the change of pure technical efficiency ($PTEC_{t}^{t+1}$) and the change of scale efficiency ($SEC_{t}^{t+1}$), $ML_{t}^{t+1}$ index is finally decomposed into Equation (6), according to which the driving factors of green development efficiency can be analyzed.

(6) $$\begin{array}{ll} ML_{t}^{t+1} & =TC_{t}^{t+1}\times PTEC_{t}^{t+1}\times SEC_{t}^{t+1}\\ & ={\left[\frac{1+\vec{D_{c}^{t+1}}(k^{t},l^{t},e^{t},y^{t},b^{t};g^{t})}{1+\vec{D_{c}^{t}}(k^{t},l^{t},e^{t},y^{t},b^{t};g^{t})}\times \frac{1+\vec{D_{c}^{t+1}}(k^{t+1},l^{t+1},e^{t+1},y^{t+1},b^{t+1};g^{t+1})}{1+\vec{D_{c}^{t}}(k^{t+1},l^{t+1},e^{t+1},y^{t+1},b^{t+1};g^{t+1})}\right]}^{\frac{1}{2}}\\ & \times \frac{1+\vec{D_{v}^{t}}(k^{t},l^{t},e^{t},y^{t},b^{t};g^{t})}{1+\vec{D_{v}^{t+1}}(k^{t+1},l^{t+1},e^{t+1},y^{t+1},b^{t+1};g^{t+1})}\\ & \times [\frac{1+\vec{D_{c}^{t}}(k^{t},l^{t},e^{t},y^{t},b^{t};g^{t})}{1+\vec{D_{v}^{t}}(k^{t},l^{t},e^{t},y^{t},b^{t};g^{t})}\times \frac{1+\vec{D_{v}^{t+1}}(k^{t+1},l^{t+1},e^{t+1},y^{t+1},b^{t+1};g^{t+1})}{1+\vec{D_{c}^{t+1}}(k^{t+1},l^{t+1},e^{t+1},y^{t+1},b^{t+1};g^{t+1})}] \end{array}$$

Among them, ${\vec{D}}_{c}$ and ${\vec{D}}_{v}$ represent the directional distance function under CRS and VRS conditions. If $TC_{t}^{t+1}>1$, it indicates that the decision-making unit has technological progress from period t to period t+1. $PTEC_{t}^{t+1}>1$, it shows that there is “catch-up effect” from period t to period t+1, that is, the production efficiency of the decision-making unit is improved. $SEC_{t}^{t+1}>1$, and it shows that the scale efficiency of the decision-making unit is improved from period t to period t+1, which is closer to the optimal scale, and vice versa.

### 3.2. Relevant Data Processing

Measuring green development efficiency also needs to set input variables and output variables and, with reference to the method of Zhang et al. [17], the capital stock, labor and energy are taken as input factors, the gross domestic product (GDP) is taken as expected output, and the waste water, waste gas and general solid waste produced in the industrial production process are taken as unexpected output. The data of each variable are processed as follows:

Factor inputs: Capital stock (k)—capital investment is the fund source of green development. The direct result of capital investment is the increase of total fixed assets, and fixed assets account for the highest proportion of capital investment in China. In view of the availability of data, we use the fixed asset investment of the whole society in the current period to measure the capital stock, and use the fixed asset investment price index to reduce it to the constant price in 1997; Labor (l)—labor input is the input level of human resources in the process of green development. According to the classical economic growth theory, labor is the core element of economic growth, so the measurement of green development efficiency must include labor input variables. We choose the number of employees in the whole society at the end of that year to measure labor input. Energy (e)—some studies use energy consumption per unit of GDP to measure regional energy efficiency, but this method ignores the structural differences and dynamic characteristics of energy utilization between regions, in order to ensure the consistency of data, we use the energy consumption of the whole society to measure energy input.

Expected output (y)—expected output is an indicator to measure the economic goal of green development. Green development is a development model based on economic growth, and the level of economic output is still the key to determine the efficiency of green development. In addition, economic growth is also the main goal pursued by local governments in China. Therefore, in this paper, the GDP of the region in the current year is chosen as the expected output, and it is converted into the real GDP at the constant price in 1997.

Unexpected output (b)—unexpected output is an important feature of green development which is different from traditional development mode. At present, China’s environmental pollution mainly comes from the industrial industry, and industrial pollution is the main source of environmental pollution. Considering the availability and integrity of the data, and drawing on the existing literature [42], this paper chooses industrial waste water, industrial waste gas and general solid waste as unexpected output.

### 3.3. Measurement Results and Analysis

#### 3.3.1. Green Development Efficiency Analysis

**Figure 2:**
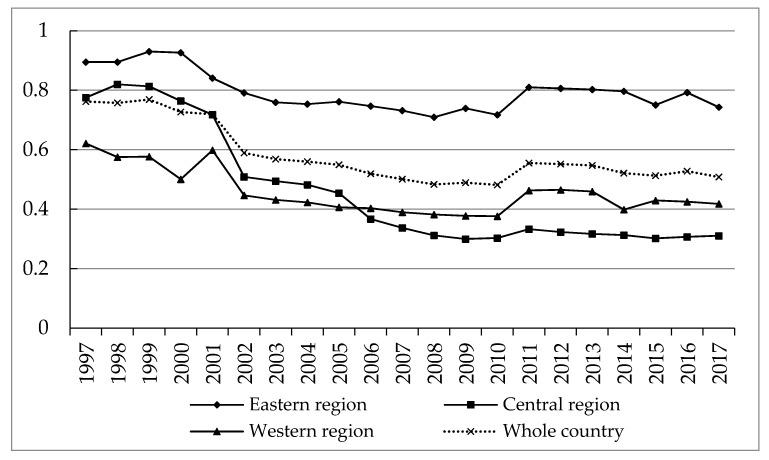
Change trend of China’s regional green development efficiency in 1997–2017.

Based on the above method, we measured the efficiency of green development in Chinese provinces from 1997 to 2017. Table 1 shows the average green development efficiency of each province, and Figure 2 shows the change trend of green development efficiency in the whole country and the eastern, central and western regions from 1997 to 2017.

In general, China’s regional green development efficiency shows the following two significant characteristics. Firstly, from the perspective of the whole country, the efficiency of national green development fluctuated and declined from 1997 to 2017. The efficiency value remained above 0.5 in most years, and only fell below 0.5 in 2008–2010. This may be due to the impact of the financial crisis on China’s economy in 2008, and the GDP growth rate in 2008 fell to 10.1% from 14.7% in 2007 (GDP growth in 2009 and 2010 was 8.5%, 10.3%). This shows that the efficiency distribution of China’s green development has certain synchronization with economic development, which is consistent with the research results of Huang et al. [43] and Yang et al. [44]. In 2010–2011, the efficiency of green development rapidly climbed to the level of 0.5554, and has been maintained above 0.5 since then. This is because the Chinese government has adopted a series of loose fiscal and monetary policies to stimulate the economy in response to the financial crisis. Overall, however, the downward trend of China’s green development efficiency has not changed since 1997, which indicates that the long-term high growth of China’s economy has led to the continuous increase of the burden on ecological environment, and the problem of resource consumption and environmental pollution has become increasingly prominent. Secondly, from the perspective of regions, the annual mean of green development efficiency in the eastern, central and western regions is quite different. The eastern region is about 0.8, while the western region is about 0.4, with significant polarization [45,46].

From 1997 to 2017, the change trend of green development efficiency in the three regions was basically the same, all of which showed a small increase in the previous years and then gradually decreased, showing a downward trend of fluctuations. Among them, the central region declined the most, from 0.7752 in 1997 to 0.3104 in 2017. Before 2006, the green development efficiency of the central region was higher than that of the western region, but after 2006, it fell to the lowest in three regions. To summarize, the green development efficiency of the eastern region was higher than that of other regions in 1997–2017, and was above the national average level. The green development efficiency of the central region was slightly higher than the national level before 2001, but lower than the national level in other years, while the western region was always below the national level. The possible reason is that the economic development of the central and western regions mainly depends on the accumulation of factors and the pull of investment. A large amount of capital is concentrated in industry. To a certain extent, the deepening of capital leads to the tendency of heavy industry in economic development, which is not conducive to the improvement of green development efficiency.

At the provincial level, the green development efficiency of the eastern provinces ranks at the top all year round, and this indicates that the green development efficiency of the eastern regions is relatively stable. Beijing, Shanghai and Guangzhou are the three economically strong provinces in China, its green development efficiency are all at the forefront. This may be because Beijing, Shanghai and Guangzhou have the highest level of economic development, which promotes the efficiency of green development to some extent, and the three regions have the highest degree of openness, and they are the gathering place of advanced technology and talents. Moreover, the transfer and elimination of polluting enterprises in recent years has optimized the industrial structure, which has a positive impact on the improvement of green development efficiency. It is worth noting that Hainan and Qinghai rank in the top five in terms of green development efficiency, both of which are at the forefront of production. The reason is that Hainan province mainly develops tourism, while Qinghai province mainly focuses on animal husbandry and tourism. The industrial structure endowments determine that there are fewer pollution enterprises in its territory, so the green development efficiency is relatively high. In contrast, the efficiency of green development in the central region is relatively low, especially in Shanxi province, which ranks the lowest in the central region. Shanxi is a large coal resource province in China. Since the reform and opening up, in order to support the national economic development, Shanxi has delivered 10 billion tons of coal to the country, and through coal power generation delivered to many provinces in China. However, the long-term resource exploitation has caused seriously environmental pollution, resulting in its green development efficiency ranking backward. The western region has the weakest economic strength and low scientific and technological content of production. While achieving the same economic benefits, it will consume more resources, resulting in low efficiency of green development. In recent years, the western region mainly undertakes the industrial transfer from the eastern region. These industries are often of high energy consumption and pollution, which seriously restricts the green development of the western region.

#### 3.3.2. Driving Factors of Green Development

We use ML index decomposition method to decompose the TFP index of regional green development in China, and analyze the driving factors of China’s regional green development efficiency from three aspects of technological progress, pure technological efficiency and scale efficiency. We obtained the TFP index and its decomposition of 30 provinces and 21 years in China from 1997 to 2017. The results are shown in Table 2 and Table 3.

At the national level, the TFP index of China’s green development efficiency in 1997–2017 is 1.0193, and it is greater than 1 in most years, which indicates that China’s green TFP in the sample period has a growth trend and strong stability. However, from the decomposition results, it is found that technological progress has increased by 6.04%, while pure technological efficiency and scale efficiency have negative growth, with growth rates of −1.14% and −0.35%. This shows clearly that China has made great progress in technological progress, which is the key factor to promote green development, but the pure technological efficiency has become the bottleneck restricting green development. At the regional level, the TFP index of the eastern and western regions are greater than 1, which are 1.0504 and 1.0107. In eastern regions, only Hebei has a TFP value less than 1, while Sichuan, Guizhou, Yunnan and Gansu in western regions have a TFP value less than 1, indicating that the green development efficiency of these two regions is increasing. Technological progress contributed the most to the efficiency of green development in these two regions, with the growth rate reaching 9.47% and 4.65%. Scale efficiency seriously restricts the green development of the eastern region, while pure technological efficiency is the restriction factor of the western region. The TFP index of central region is 0.9844, which is the only region showing a downward trend. The reason is that the pure technical efficiency has declined significantly, reaching −3.05%.

Furthermore, the driving factors of green development efficiency are analyzed. Firstly, the role of technological progress in promoting the efficiency of green development in the three regions gradually decreased from the eastern region, the western region to the central region, and the technological progress indexes were all greater than 1. This is largely due to China’s vigorous promotion of scientific and technological innovation in recent years. For example, the R&D investment increased from $0.56 million in 1997 to $17.79 million in 2017. Obviously, China has made remarkable achievements in strengthening investment of technological innovation, and technological progress brought by technological innovation has become a strong driving force for green development. Secondly, the pure technical efficiency index of the three regions were all less than 1, and the central region was the lowest, which indicates that the three regions, especially the central region, are in urgent need of improving the resource allocation and production efficiency, which is also a key issue for China to further promote green development. Finally, scale efficiency showed significant regional difference. The scale efficiency index in the eastern region was less than 1, while those in the central and western regions were greater than 1, indicating that the eastern region is in the stage of diseconomies of scale. The possible reason is that for a long time, the factors such as energy, capital, and talents have been excessively concentrated in the eastern region, which has exceeded the optimal scale, leading to the reduction of scale returns. Therefore, the eastern region should pay more attention to technological innovation rather than expanding production scale.

## 4. Econometric Model Variable Setting and Data Processing

### 4.1. Mediating Effect Model

Under China’s current economic decentralization system, although local government has certain economic autonomy and can adjust the financial expenditure and investment structure according to the development goals of the jurisdiction, central government has an absolute discourse power in the political career of local government officials. Economic performance is a key indicator for evaluating the performance of officials at all levels. In addition, the tenure of local government officials is generally 5 years, and most of them are transferred from other places, which leads to local governments pay more attention to short-term economic benefits. The dual incentive of politics and economy urges local governments to carry out strategic competition in all aspects.

First of all, local governments that pursue high GDP growth are bound to vigorously develop secondary industries, which contribute to faster economic growth. However, the secondary industry needs to invest a lot of natural resources in the production process and produce more industrial pollutants. But in consideration of their own career, the local governments fall into the irrational competition for GDP growth. In this process, on the one hand, local governments are more inclined to reduce the level of local environmental regulation to attract industrial enterprises to produce and operate in the local area. On the other hand, environmental regulation will lead to an increase in the “compliance cost” of enterprises’ production. In order to gain competitive advantage, enterprises will also choose the areas with lower standards of environmental regulation to invest, forming a “pollution refuge” [35], which is not conducive to the green development of this region. Secondly, ecological environment is a typical non-economic public product. Local governments tend to have the mentality of “free rider” and lack the motivation of environmental governance. Therefore, environmental regulation level is reduced to reduce the fiscal expenditure of environmental governance, which is not conducive to the green development of this region. In addition, technology research and development (R&D) expenditure will stimulate technological innovation of enterprises, greatly improve the efficiency of resource utilization, accelerate industrial upgrading and promote economic growth in the long run, so as to play a positive role in promoting regional green development. However, science and technology innovation projects often have the characteristics of high investment, high risk and long cycle, and will crowd out other infrastructure investment in the short term, so they are not favored by local governments. The fiscal expenditure structure of local governments, which emphasizes economic infrastructure, ignores science and technology innovation and environmental governance, distorts the allocation of regional resources and is not conducive to the improvement of regional green development efficiency. Thirdly, the competition of foreign direct investment (FDI) by local governments is the main representation of investment competition. In the early stage of competition, local governments attract FDI to introduce advanced production technology and management experience by reducing the level of environmental regulation, which not only has a demonstration effect on local enterprises, but also increases the competitive pressure of local enterprises, promotes local enterprises to speed up technological innovation [47], and contributes to the improvement of green development efficiency. However, with the continuous influx of FDI and the continuous expansion of production scale, which brings huge energy consumption and the increase of unexpected output, local governments tend to have a higher level of environmental regulation to stimulate the innovation willingness of enterprises in green technology [48], which plays a positive role in promoting the local green development.

To sum up, China’s local government competition involves many aspects, but whether it is growth competition, fiscal competition or investment competition, it is inseparable from the government’s choice of environmental regulation level. That is, environmental regulation has become a transmission variable of local government competition affecting green development efficiency. Therefore, this paper uses Baron and Kenny’s [49] mediating effect model for reference, takes environmental regulation as mediating variable to test the effect of local government competition on green development efficiency, which is realized by the following model:

Step 1: Regress local government competition and green development efficiency, and build the following benchmark regression model. If the local government competition coefficient is significant, it indicates that local government competition can affect green development efficiency, step 2 can be conducted.

(7) $$gde_{it}=\beta_{0}+\beta_{1}gc_{it}+\beta_{p}P_{it}+\mu_{i}+\nu_{t}+\varepsilon_{it}$$

In Equation (7), i represents the section element and t represents the time element. gde is the efficiency of regional green development, gc is the core explanatory variable of this paper, local government competition, including growth competition (gcgdp), fiscal competition (gcfre) and investment competition (gcfdi). P represents the set of control variables that may affect the efficiency of green development in addition to local government competition, $\mu_{i}$ is the regional effect, $\nu_{t}$ is the time effect, $\varepsilon_{it}$ is random disturbance term. β is the regression coefficient, and $\beta_{1}$ is the core coefficient that this paper focuses on, that is, the impact of local government competition on green development efficiency.

Step 2: Introduce mediating variables ($MP_{it}$) to conduct regression of local government competition, and test whether local government competition affects mediating variables; the model is Equation (8):

(8) $$MP_{it}=\alpha_{0}+\alpha_{1}gc_{it}+\alpha_{p}P_{it}+\mu_{i}+\nu_{t}+\varepsilon_{it}$$

In the above equation, MP is the mediating variable, i.e., environmental regulation (erl), α is the regression coefficient, if $\alpha_{1}$ is significant, it indicates that local government competition has an impact on environmental regulation, and the next step can be taken.

Step 3: After the introduction of mediating variables, regress local government competition and green development efficiency again; the model is Equation (9):

(9) $$gde_{it}=\beta_{0}+\beta_{1}gc_{it}+\beta_{2}MP_{it}+\beta_{p}P_{it}+\mu_{i}+\nu_{t}+\varepsilon_{it}$$

In the above equation, on the premise that the regression coefficient (*β*_2_)of mediating variable is significant, if the regression coefficient (*β*_1_) of local government competition is smaller and still significant, it indicates that the influence of local government competition on green development efficiency partly comes from mediating variables; If the regression coefficient (*β*_1_) of local government competition becomes insignificant, it shows that the influence of local government competition on green development efficiency is entirely from mediating variables.

### 4.2. Variable Setting

#### 4.2.1. Core Independent Variable and Intervening Variable

Local government competition (gc): Local government competition is the core variable that this paper focuses on. Through the previous analysis, China’s local government competition involves many aspects. This paper measures the competitiveness of local government from three perspectives: growth competition, fiscal competition and investment competition. Growth competition (gcgdp): under the evaluation mechanism of Chinese government officials, economic growth is related to the evaluation and promotion of local government officials, which has become the core goal of local government competition. So we use the annual growth rate of regional GDP to measure the growth competition, which reflects the competitiveness brought by the growth incentives of local governments. Fiscal competition (gcfre): the institutional arrangement of Chinese decentralization enables local governments to influence regional economic development by controlling the investment scale and direction of public finance. The more financial expenditure used for environmental governance, the better the efficiency of green development. So we use the financial self-sufficiency rate of local government to measure fiscal competition, that is, the ratio of fiscal expenditure and fiscal revenue of local governments. The larger the ratio, the stronger the competitiveness of local governments. Investment competition (gcfdi): FDI often has a high technology content, which is conducive to the learning and re-innovation of enterprises. To a certain extent, it can improve the local technology level, so as to promote the efficiency of regional green development. Therefore, the competition of local governments for FDI is a concentrated reflection of investment competition. We use the logarithmic value of regional per capita actual utilization of FDI to calculate the investment competition. The larger value indicates the stronger the competitiveness of local governments in the region.

Environmental regulation (erl): environmental regulation includes formal and informal. The object of this paper is local government, and the behavior choice of local government mainly affects the formal environmental regulation. Formal environmental regulation means that the government regulates market economic activities by formulating corresponding policies, and achieves the coordination between economic growth and environmental protection by preventing and controlling industrial pollution and protecting the ecological environment. This paper focuses on the investment of local governments in environmental pollution control, and chooses the ratio of total investment in regional environmental pollution control to regional GDP to measure the level of environmental regulation. The larger value indicates the higher the level of environmental regulation in this region.

#### 4.2.2. Control Variables

Marketization (mar): the higher the marketization level is, the better the spillover effect of technology will be brought into play, thus contributing to the improvement of green development efficiency. China’s market-oriented reform involves all-round changes in society, economy and law in the process of economic system transformation. The research group of China’s market-oriented index has explored the internal mechanism and influencing factors of the market-oriented process in depth, and comprehensively measured China’s market-oriented index according to five aspects, including the relationship between government and market, the development of non-state-owned economy, the development of product market, the development of factor market, the development of market intermediary organization and rule of law environment. This paper uses the marketization index measured by the research group.

Technological level (tec): technological progress is conducive to improving the efficiency of green development, especially with the application of green technology, it can achieve a green economic growth model with low energy consumption and low pollutant emission while producing high output. This paper uses the natural logarithm of the number of patent applications authorized in that year to measure technical level.

Industrial structure (str): with the development of information technology, “economic servitization” has become a trend of industrial structure change, which helps to improve the efficiency of green development. Therefore, this paper adopts the non-agricultural index of industrial structure, that is, the ratio of output value of secondary and tertiary industry to GDP.

Openness to the outside world (open): since the reform and opening up, China’s economy has been an full-opened outward-looking economy. Opening up to the outside world affects the inflow of international advanced technology, and its spillover effect affects the local ecological environment and resource dependence to a certain extent. In this paper, the ratio of regional total import and export trade to GDP is used to measure the level of opening up.

Human capital stock (hum): the promotion of human capital can improve the scientific and technological level of labor force, promote the improvement of labor productivity, and produce a crowding out effect on polluting technology. In addition, with the general improvement of human capital level in the whole society, the public’s awareness of environmental protection is becoming increasingly strong, which tends to a greener lifestyle, so as to promote the efficiency of green development. In this paper, the average education years of the population over 6 years old is adopted to measure the level of human capital, we determine the weight according to the general length of schooling at all levels of school education in China. The specific calculation formula is as follows:

Human capital = (Illiterate population×0 + Primary education population × 6 + Junior middle school education population × 9 + High school education population × 12 + Population with college education or above × 16)/Total population over 6 years old(10)

Capital stock per capita (cap): according to the Rybczynski theorem, when the capital stock per capita increases, the output of capital intensive enterprises will increase, thus increasing the emission of pollutants, which is not conducive to the improvement of green development efficiency. Therefore, this paper chooses the natural logarithm of capital formation per capita in each province to measure the capital stock per capita.

### 4.3. Data Description

The empirical part of this paper selects the provincial panel data of 30 provinces (there are a lack of data for Tibet, Hong Kong, Macao and Taiwan) in mainland China from 1997 to 2017. All the data are collected from China Statistical Yearbook, China Fixed Asset Investment Statistical Yearbook, China Energy Statistical Yearbook, China Science and Technology Statistical Yearbook, and the statistical yearbooks of all provinces (autonomous regions and municipalities directly under the Central Government). Some missing data are supplemented by the interpolation method. In addition, in order to eliminate the interference of heteroscedasticity and dimensional problems, the absolute value data are logarithmically processed. The descriptive statistics of each variable are shown in Table 4.

As we can see, each variable includes 30 cross sections and 630 observations. The results show that the mean value of gde is 0.5811, the median value is 0.4122, the maximum value is 1.0000, and the minimum value is 0.2198, the standard deviation is 0.3003, indicating that the efficiency of green development in the observation period has a large variation, and other variable sequences also have values covering a wide range, which provides sufficient information for this paper to analyze the relationship between local government competition and regional green development in China.

## 5. Empirical Results and Discussion

### 5.1. Benchmark Regression Results

Through the Hausman test, this paper adopts the panel fixed effect model to test the effect of three types of local government competition on green development efficiency. Table 5 shows the estimation results based on the benchmark regression model (7).

Model (1), (3) and (5) are estimated results excluding control variables, while model (2), (4) and (6) are estimated results after adding control variables. All models considered time and regional effect. It can be found that the regression coefficient of local government competition in all models are significantly negative at the level of 1%, indicating that no matter what type of local government competition, it has a restraining effect on green development efficiency. In order to accelerate the economic development of the region and improve the performance of local government, the competition policies formulated by local government will reduce the green development efficiency of the region to some extent. It can be seen from models (2), (4) and (6) that the influence coefficient of marketization, openness to the outside world and capital stock per capita on green development efficiency is negative, and the significance level is different between models, indicating that marketization, openness to the outside world and capital stock per capita are not conducive to the improvement of China’s green development efficiency. The impact of technological innovation and human capital stock on green development efficiency is positive, meeting the significance level test of at least 10%, indicating that the improvement of technological innovation and human capital stock has a strong promoting effect on green development efficiency. The industrial structure has a positive effect on green development efficiency, but it is not statistically significant.

### 5.2. Discussion on Endogenous and Robustness

The dynamic nature of economic development makes economic variables often have the characteristics of path dependence. At the same time, considering that there may be a two-way impact between local government competition and green development efficiency, thus generating endogenous problems. The generalized moment estimation (GMM) increases lag term of the explained variable as a tool variable, and solves dynamic and endogenous problems. Therefore, this paper adopts the dynamic panel difference (DIF) GMM model to test the estimation results above. Models (1)–(3) in Table 6 report the estimated results. AR (2) results show that there is no high-order autocorrelation in the residual sequence, and Sargan test results show that the tool variable setting meets the effectiveness requirements. Model (1) shows that although the estimated coefficient of local government growth competition is positive, it is not statistically significant. Model (2) shows that the regression coefficient of local government fiscal competition is negative and highly significant at the level of 1%. Model (3) shows that the estimated coefficient of local government investment competition is significantly negative at the level of 5%. This shows clearly that after alleviating the endogenous problems, the impact of local government competition on green development efficiency is still negative, and local government competition is not conducive to the improvement of green development efficiency. In addition, the estimation coefficient of the explained variable with one lag period (L.gde) indicates that the green development efficiency has a significant path-dependent feature, and the influence is significantly positive at the level of 1%, indicating that green development efficiency is affected by the initial level.

The benchmark regression results may be affected by the estimation methods, and the green development efficiency belongs to the interception data. Therefore, in order to test the robustness of the estimation results, the panel data Tobit model is adopted in this paper for the robustness test. Models (4)–(6) in Table 6 report the estimated results. It is not difficult to find that the regression coefficient of three types of local government competition to green development efficiency is still negative, and at least meets the significance test of 5%, which is consistent with the benchmark regression analysis conclusion. This shows that the inhibition effect of local government competition on green development efficiency is very stable, and the main conclusions of this paper are reliable.

### 5.3. The Test of Mediating Effect Based on Environmental Regulation

The impact of local government competition on green development efficiency in China is largely achieved by choosing the level of environmental regulation. This paper further takes environmental regulation as an mediating variable to test the mechanism of local government competition affecting green development efficiency. Table 7 reports the regression results of mediating effect. Models (2), (4) and (6) in Table 5 are the regression results of the first step based on local government growth competition, fiscal competition and investment competition. Models (1), (3) and (5) in Table 7 are the second step regression results based on local government growth competition, fiscal competition and investment competition, and models (2), (4) and (6) are corresponding regression results of the third step.

Firstly, model (1) shows that the regression coefficient of local government growth competition to environmental regulation is not significant, but t value is greater than 1, indicating that local government growth competition has a weak positive effect on the level of environmental regulation. It can be seen from model (2) that the regression coefficient of local government growth competition is negative and significant, which once again verifies that local government growth competition will inhibit the improvement of green development efficiency, but the regression coefficient of environmental regulation is not significant, which indicates that environmental regulation does not inhibit the green development efficiency. Therefore, the empirical results do not support the mediating effect of environmental regulation on local government growth competition, that is, there is no mediating effect of local government growth competition on green development efficiency. Secondly, model (3) shows that the impact coefficient of local government fiscal competition on environmental regulation is significantly negative at the level of 1%, which indicates that fiscal competition will indeed lead to the reduction of environmental regulation level. From model (4), it can be found that the regression coefficient of environmental regulation is significantly negative at the level of 1%, while the regression coefficient of fiscal competition is still negative and highly significant after adding mediating variables, but the absolute value changes from 0.1258 to 0.1140, which weakened the effect. This shows that under the fiscal competition of local government, environmental regulation has partial mediating effect. The fiscal competition of local government restrains green development efficiency by reducing the level of environmental regulation. Finally, model (5) shows that the impact coefficient of local government investment competition on environmental regulation is positive and meets the significance test of 1%. It can be found from model (6) that the regression coefficient of environmental regulation is significantly negative at the level of 10%, and the impact of investment competition on green development efficiency is significantly negative at the level of 1%, but the absolute value of the impact changes from 0.0517 to 0.0496, the impact intensity is reduced. This indicates that under the investment competition of local government, there are some mediating effects in environmental regulation. That is, local governments compete to reduce the level of environmental regulation in order to introduce foreign direct investment, which is not conducive to the improvement of green development efficiency in the region.

## 6. Conclusions

This paper measures the green development efficiency of 30 provinces in China from 1997 to 2017 by using the total-factor non-radial directional distance function and the SBM-DEA model. This paper also decomposes the green development efficiency into technical progress, pure technical efficiency and scale efficiency based on the ML index method. Then, based on China’s provincial panel data from 1997 to 2017, taking environmental regulation as a mediating variable, this paper empirically analyzes the influence mechanism of local government competition on green development efficiency from three perspectives including growth competition, fiscal competition and investment competition. The main conclusions suggested that the efficiency of regional green development in China showed a fluctuating downward trend, and the efficiency value remained above 0.5 from 1997 to 2017. At the regional level, the green development efficiency of the eastern, central and western regions all showed a small increase in the previous years, and then gradually decreased. There are significant regional differences, the eastern region had the highest green development efficiency, and before 2006, the green development efficiency of the central region was higher than that of the western region, but after 2006, it fell to the lowest in three regions. The TFP index decomposition results show that technological progress is the key factor to promote green development, and its role in promoting green development efficiency in the three regions gradually decreases from the eastern region to western region and central region. Pure technical efficiency has become a bottleneck restricting the improvement of green development efficiency, while scale efficiency shows significant regional differences. The eastern region is in the stage of diseconomies of scale, while the central and western regions are in the stage of economies of scale. Additionally, the empirical results show that the growth competition, fiscal competition and investment competition of local government all have a significant inhibitory effect on the efficiency of green development. Among them, the impact of fiscal competition and investment competition on the efficiency of green development have some mediating effects, that is, they restrain green development efficiency by reducing the level of environmental regulation. However, growth competition has no mediating effect on green development efficiency.

Among the Sustainable Development Goals (SDGs), there are seven goals that are directly related to the green development mode, and the conclusions of this paper are not only of great significance to the policy making of local governments in promoting regional green development in China, but also of great reference value to other countries to achieve sustainable development goals. Firstly, the government should increase its support for technology research and development of enterprises, especially green technology research and development, encourage enterprises to improve energy utilization efficiency and reduce pollution emissions through technological innovation, and accelerate the construction of market-oriented green technology innovation system to promote the efficiency of China’s green development through technological progress. It should encourage and guide enterprises and scientific research institutes to deepen exchanges and cooperation, and accelerate the market-oriented application of technological achievements, guide local governments, private sector and civil society organizations to establish partnerships for sustainable development. At the same time, it should strengthen exchanges between developed and developing countries, and the eastern, central and western regions, promote the flow of talents, technologies and other factors of production around the world, so as to better exert the spillover effect of advanced technology, and achieve the goal of sustainable development. Secondly, it should further improve the efficiency of resource allocation and increase the proportion of resources allocated by the market. The eastern region should improve the situation of excessive concentration of capital, energy and other factors of production, and further optimize the management level. The central and western regions should develop new energy such as wind energy and solar energy in combination with the local natural resource endowment, improve the utilization efficiency of renewable energy, and reduce the carbon intensity of energy, then improve the level of environmental pollution control in the process of undertaking the industrial transfer in the eastern region, to gradually reverse the low efficiency of green development. Thirdly, the central government should strengthen the institutional norms and incentive constraints for the behavior choice of local governments, and incorporate pollution control and ecological environment protection into the local government performance appraisal system and the incentive system for the promotion of officials. It should gradually carry out the audit of officials leaving their posts for the construction of ecological civilization, and guide the establishment of a green development-oriented competition mechanism for local governments. Furthermore, government should create a good atmosphere for green competition among local governments to promote the efficiency of green development. Finally, the government should set up natural asset management and ecological environment supervision institutions, and improve the ecological environment supervision system. It should guide and encourage social capital to enter the fields of pollution control and environmental protection, enhance public awareness of environmental protection and form a sustainable consumption and production model. It should introduce a market mechanism in the field of environmental regulation, and give full play to the positive role of informal environmental regulation to promote the further improvement of green development efficiency. It should give all people access to basic public services, green and decent jobs, improved quality of life, and achieve a sustainable development model.

Although this paper reveals the impact of local government competition on regional green development in China, and obtains some valuable conclusions, due to the data and analysis perspective other factors affecting regional green development cannot be analyzed in depth; nor can the paper provide a complete plan for other countries to achieve green development. Future research can be expanded in the following two aspects: (1) considering the spatial effect of local government competition, the impact on green development needs to be explored from a spatial perspective. (2) This paper explores regional green development from the perspective of the government, without considering the impact of enterprises, families and individuals on green development, which will be the focus of future research.

## Figures and Tables

**Table 1:** Average efficiency of green development in China’s provinces in 1997–2017.

Region	Mean Value	Ranking	Region	Mean Value	Ranking	Region	Mean Value	Ranking
Beijing	1.0000	1	Fujian	0.6431	11	Xinjiang	0.3872	21
Shanghai	1.0000	2	Heilongjiang	0.6232	12	Hubei	0.3825	22
Guangdong	1.0000	3	Inner Mongolia	0.5721	13	Chongqing	0.3592	23
Hainan	1.0000	4	Liaoning	0.5318	14	Hebei	0.3450	24
Qinghai	1.0000	5	Jiangxi	0.4932	15	Shaanxi	0.3409	25
Tianjin	0.9321	6	Hunan	0.4840	16	Sichuan	0.3276	26
Jiangsu	0.8203	7	Jilin	0.4676	17	Shanxi	0.3238	27
Shandong	0.7896	8	Henan	0.4653	18	Gansu	0.3199	28
Zhejiang	0.6841	9	Guangxi	0.4569	19	Yunnan	0.3122	29
Ningxia	0.6531	10	Anhui	0.4369	20	Guizhou	0.2804	30

**Table 2:** TFP index of China’s provinces in 1997–2017 and its decomposition.

Province	TFP	Technical Progress	Pure Technical Efficiency	Scale Efficiency
Beijing	1.1524	1.1524	1.0000	1.0000
Tianjin	1.0914	1.1650	1.0109	0.9897
Hebei	0.9961	1.0371	0.9724	0.9937
Liaoning	1.0210	1.0720	0.9666	0.9958
Shanghai	1.1097	1.1097	1.0000	1.0000
Jiangsu	1.0670	1.0881	1.0383	0.9705
Zhejiang	1.0493	1.0891	0.9906	0.9771
Fujian	1.0087	1.0649	0.9587	0.9934
Shandong	1.0143	1.0904	1.0437	1.0323
Guangdong	1.0132	1.0536	1.0000	0.9651
Hainan	1.0309	1.1191	1.0000	0.9464
Eastern Region	1.0504	1.0947	0.9983	0.9876
Shanxi	1.0133	1.0315	0.9926	0.9904
Jilin	0.9924	1.0437	0.9564	1.0100
Helongjiang	0.9549	1.0440	0.9505	1.0038
Anhui	0.9553	1.0048	0.9689	1.0030
Jiangxi	0.9798	1.0422	0.9490	1.0180
Henan	1.0029	1.0283	0.9976	0.9967
Hubei	1.0041	1.0336	0.9855	0.9919
Hunan	0.9726	1.0299	0.9557	0.9918
Central Region	0.9844	1.0323	0.9695	1.0007
Inner Mongolia	1.0571	1.1140	1.0447	1.0949
Guangxi	1.0099	1.0523	0.9803	1.0095
Chongqing	1.0103	1.0359	0.9779	1.0026
Sichuan	0.9937	1.0280	0.9783	0.9921
Guizhou	0.9938	1.0199	0.9782	0.9978
Yunnan	0.9877	1.0284	0.9710	0.9922
Shaanxi	1.0134	1.0378	0.9791	1.0018
Gansu	0.9959	1.0242	0.9989	0.9769
Qinghai	1.0180	1.0547	1.0000	0.9655
Ningxia	1.0250	1.0546	1.0384	1.0091
Xinjiang	1.0135	1.0621	0.9726	0.9830
Western Region	1.0107	1.0465	0.9927	1.0023
Whole Country	1.0183	1.0604	0.9886	0.9965

**Table 3:** Annual TFP index and its decomposition in 1997–2017.

Time Interval	TFP	Technical Progress	Pure Technical Efficiency	Scale Efficiency	Time Interval	TFP	Technical Progress	Pure Technical Efficiency	Scale Efficiency
1997–1998	0.9478	0.9450	0.9847	1.0276	2007–2008	1.0782	1.1838	0.9651	0.9790
1998–1999	1.0784	1.0724	1.0244	0.9867	2008–2009	0.9609	0.9975	0.9994	0.9698
1999–2000	1.0588	1.0779	0.9594	1.0475	2009–2010	1.0758	1.0897	0.9955	0.9968
2000–2001	1.0351	1.1754	1.0172	0.9290	2010–2011	0.7067	0.6522	1.1911	0.9593
2001–2002	0.9617	1.0725	0.8637	1.1112	2011–2012	1.0307	1.0628	0.9875	0.9831
2002–2003	0.9818	1.0012	0.9642	1.0296	2012–2013	1.0705	1.1174	0.9857	0.9740
2003–2004	1.0368	1.0549	0.9790	1.0048	2013–2014	1.0285	1.0748	0.9638	1.0229
2004–2005	0.9896	1.0588	0.9877	0.9685	2014–2015	1.0250	1.0670	0.9856	0.9929
2005–2006	1.0346	1.1041	0.9507	0.9911	2015–2016	1.1007	1.1234	1.0248	0.9668
2006–2007	1.0688	1.1260	0.9567	0.9925	2016–2017	1.0948	1.1507	0.9852	0.9971
Mean Value	1.0183	1.0604	0.9886	0.9965					

**Table 4:** Descriptive statistics of variables.

Variables	gde	gcgdp	gcfre	gcfdi	erl	mar	tec	str	open	hum	cap
Mean	0.5811	0.1311	1.9795	5.9858	1.1692	5.1101	8.5105	0.8780	0.2996	8.4473	9.1901
Median	0.4122	0.1211	2.0450	6.1356	1.0761	4.8873	8.4141	0.8798	0.1252	8.4607	9.3355
Maximum	1.0000	0.3227	6.3057	9.0489	4.2314	10.0000	12.7149	0.9964	1.6985	12.5025	11.2807
Minimum	0.2198	−0.2240	0.5577	2.1527	0.0038	1.1030	4.0254	0.6532	0.0164	4.6926	6.7749
Std.Dev.	0.3003	0.0620	0.8200	1.4297	0.6775	1.9388	1.7234	0.0640	0.3711	1.1011	1.0409
Skewness	0.5224	0.0621	0.6249	−0.2934	1.3384	0.4787	0.0895	−0.4127	1.9906	0.3003	−0.1796
Kurtosis	1.4956	4.4086	3.7014	2.3227	5.8606	2.6844	2.6228	3.5958	6.1476	4.0310	1.8918
Jarque–Bera	88.0617	52.4860	53.9108	21.0813	402.9012	26.6721	4.5767	27.2043	676.1262	37.3746	35.6279
Observations	630	630	630	630	630	630	630	630	630	630	630
Cross Sections	30	30	30	30	30	30	30	30	30	30	30

**Table 5:** Benchmark regression results.

Explanatory Variable	gcgdp		gcfre		gcfdi	
Model	(1)	(2)	(3)	(4)	(5)	(6)
β_1_	−0.6040 ***	−0.4190 ***	−0.1610 ***	−0.1258 ***	−0.0975 ***	−0.0517 ***
	(−5.31)	(−3.76)	(−8.56)	(−5.92)	(−12.89)	(−4.28)
mar		−0.0304 ***		−0.0448 ***		−0.0259 **
		(−2.84)		(−4.12)		(−2.41)
tec		0.0644 ***		0.0936 ***		0.0873 ***
		(3.59)		(5.62)		(5.22)
str		0.2600		0.0975		0.3050 *
		(1.46)		(0.54)		(1.72)
open		−0.1070 *		−0.1560 ***		−0.1280 **
		(−1.96)		(−2.99)		(−2.41)
hum		0.0520 **		0.0564 ***		0.0392 *
		(2.41)		(2.59)		(1.80)
cap		−0.1390 ***		−0.1390 ***		−0.1180 ***
		(−7.35)		(−7.42)		(−5.84)
Region/Time	Yes	Yes	Yes	Yes	Yes	Yes
Adj R2	0.0449	0.2280	0.1091	0.2996	0.2171	0.2929
*N*	630	630	630	630	630	630

Notes: Numbers in parentheses are *t*-statistics for parameter estimation; * for 10% level significant, ** for 5% level significant, *** for 1% level significant.

**Table 6:** Endogenous and robustness regression results.

Method	Endogenous Discussion			Robustness Test		
	DIF-GMM			Tobit		
Model	(1)	(2)	(3)	(4)	(5)	(6)
Explanatory Variable	gcgdp	gcfre	gcfdi	gcgdp	gcfre	gcfdi
β_1_	0.0105	−0.0540 ***	−0.0070 **	−0.4940 ***	−0.1410 ***	−0.0409 **
	(0.62)	(−5.91)	(−2.00)	(−3.23)	(−5.26)	(−2.18)
L.gde	0.5820 ***	0.5650 ***	0.5730 ***			
	(35.39)	(32.30)	(43.99)			
sigma_u				0.5500 ***	0.5860 ***	0.6150 ***
				(5.63)	(5.93)	(5.91)
sigma_e				0.1870 ***	0.1830 ***	0.1870 ***
				(27.28)	(27.30)	(27.26)
AR(2)_P	1.8959 {0.0580}	1.8855 {0.0594}	1.8805 {0.0600}			
Sargan_P	23.0303 {1.0000}	24.3448 {1.0000}	23.5503 {1.0000}			
Control Variables	Yes	Yes	Yes	Yes	Yes	Yes
Region/Time	Yes	Yes	Yes	Yes	Yes	Yes
*N*	570	570	570	630	630	630

Notes: Numbers in “()” are t-statistics for parameter estimation; Numbers in “{}”are *p* values; ** for 5% level significant, *** for 1% level significant.

**Table 7:** Mediating effect regression results.

Model	(1)	(2)	(3)	(4)	(5)	(6)
Explained Variable	erl	gde	erl	gde	erl	gde
gcgdp	0.5580	−0.4060 ***				
	(1.51)	(−3.65)				
gcfre			−0.4110 ***	−0.1140 ***		
			(−6.52)	(−5.68)		
gcfdi					0.1050 ***	−0.0496 ***
					(2.63)	(−4.09)
erl		−0.0194		−0.0434 ***		−0.0221 *
		(−1.57)		(−3.45)		(−1.78)
Control Variables	Yes	Yes	Yes	Yes	Yes	Yes
Region/Time	Yes	Yes	Yes	Yes	Yes	Yes
Adj R^2^	0.1083	0.5239	0.1648	0.3134	0.1152	0.6462
*N*	630	630	630	630	630	630

Notes: Numbers in parentheses are *t*-statistics for parameter estimation; * for 10% level significant, *** for 1% level significant.

## References

[1] Babecky J., Campos N.F. (2011). Does reform work? An econometric survey of the reform-growth puzzle. J. Comp. Econ..

[2] Literature Research Office of the CPC Central Committee (2017). Xi Jinping’s Discussion Excerpt on the Construction of Socialist Ecological Civilization.

[3] Lyytim K.J., Antikainen R., Hokkanen J., Koskela S., Kurppa S., Känkänen R., Seppälä J. (2017). Developing key indicators of green growth. Sustain. Dev..

[4] Zhang J., Chang Y., Zhang L., Li D. (2018). Do technological innovations promote urban green development? A spatial econometric analysis of 105 cities in China. J. Clean. Prod..

[5] Ehresman T.G., Okereke C. (2015). Environmental justice and conceptions of the green economy. Int. Environ. Agreem. Politics Law Econ..

[6] Boulding K.E. The economics of the coming spaceship earth. Proceedings of the Sixth Resources for the Future Forum on Environmental Quality in a Growing Economy.

[7] Pearce D., Markandya A., Barbier E.B. (1989). Blueprint for a Green Economy.

[8] Zhang N., Choi Y. (2013). Environmental energy efficiency of China’s regional economies: A non-oriented slacks-based measure analysis. Soc. Sci. J..

[9] Zhang H., Feng C., Liu G.C. (2017). Chinese-style environmental federalism: A study on the effect of environmental decentralization on carbon emissions. J. Financ. Econ..

[10] Pierre-André J., Perthuis C.D. (2013). Green growth: From intention to implementation. Int. Econ..

[11] Sabit D. (2014). Green economy innovation based development of Kazakhstan. Procedia Soc. Behav. Sci..

[12] Huang M.X., Ye Q. (2017). The Marxist green development concept and green development in contemporary China: Comment on incompatibility theory between environment and development. Econ. Res. J..

[13] Wu A.H., Cao Y.Y., Liu B. (2013). Energy efficiency evaluation for regions in China: An application of DEA and Malmquist indices. Energy Effic..

[14] Qing Y., Xing Z.W., Hui M.M. (2015). Assessing green development efficiency of municipalities and provinces in China integrating models of super-efficiency DEA and malmquist index. Sustainability.

[15] Dyckhoff H., Allen K. (2001). Measuring ecological efficiency with data envelopment analysis. Eur. J. Oper. Res..

[16] Zhou P., Ang B.W., Wang H. (2012). Energy and CO_2_ emission performance in electricity generation: A non-radial directional distance function approach. Eur. J. Oper. Res..

[17] Zhang N., Kong F., Choi Y. (2014). The effect of size-control policy on unified energy and carbon efficiency for Chinese fossil fuel power plants. Energy Policy.

[18] Tone K. (2003). Dealing with undesirable outputs in DEA: A slacks-based measure (SBM) approach. GRIPS Res. Rep. Ser..

[19] Li X.X., Liu Y.M., Song T. (2014). Calculation of the green development index. Soc. Sci. China.

[20] Sun C.Z., Tong Y.L., Liu W.X. (2017). Measurement of green development level and its dynamic evolution rule in China. Econ. Geogr..

[21] Brunnermeier S.B., Cohen M.A. (2003). Determinants of environmental innovation in US manufacturing industries. J. Environ. Econ. Manag..

[22] Horbach J. (2008). Determinants of environmental innovation: New evidence from German panel data sources. Res. Policy.

[23] Langpap C., Shimshack J. (2010). Private citizen suits and public enforcement: Substitutes or complements. Environ. Econ. Manag..

[24] Cole M.A., Elliot R.J., Okubo T. (2013). The carbon dioxide emissions of firms: A spatial analysis. J. Environ. Econ. Manag..

[25] Martínez Z.I., Maruotti A. (2011). The impact of urbanization on CO_2_ emissions: Evidence from developing countries. Ecol. Econ..

[26] Li S.J., Zhang Y.M. (2015). Financial development, environmental quality and economic growth. Sustainability.

[27] Lan J., Munro A. (2013). Environmental compliance and human capital: Evidence from Chinese industrial firms. Resour. Energy Econ..

[28] Shi D. (2018). The green development and the new stage of industrialization: Progress in China and comparison with others. China Ind. Econ..

[29] Li H., Zhou L.A. (2003). Political turnover and economic performance: The incentive role of personnel control in China. J. Public Econ..

[30] Zhou L. (2017). Local Government in Transition: Official Incentives and Governance.

[31] Deng J., Zhang N., Ahmad F., Draz M.U. (2019). Local government competition, environmental regulation intensity and regional innovation performance: An empirical investigation of Chinese Provinces. Int. J. Environ. Res. Public Health.

[32] Perez S.F., Raveh O. (2016). The natural resource curse and fiscal decentralization. Am. J. Agric. Econ..

[33] Li G.L., Zhou Y.L. (2019). Environmental decentralization, local government competition and green development. Public Financ. Res..

[34] Heyes A. (2009). Is environmental regulation bad for competition? A survey. J. Regul. Econ..

[35] Porter M.E., Vander C.L. (1995). Green and competitive: Ending the statement. Harv. Bus. Rev..

[36] Millimet D. (2003). Assessing the empirical impact of environmental federalism. J. Reg. Sci..

[37] Sjöberg E., Xu J. (2018). An empirical study of US environmental federalism: RCRA enforcement from 1998 to 2011. Ecol. Econ..

[38] Stewart R.B. (1977). Pyramids of sacrifice: Problems of federalism in mandating state implementation of national environmental policy. Yale Law J..

[39] Stewart J.M. (2017). Environmental law-an environmental federalism dust-up: EPA’s authority to override unreasonable state permitting decisions under the clean air act. Wyo. Law Rev..

[40] Barros C.P., Managi S., Matousek R. (2012). The technical efficiency of the Japanese banks: Non-radial directional performance measurement with undesirable output. Omega.

[41] Chung Y.H., Fare R., Grosskopf S. (1997). Productivity and undesirable outputs: A directional distance function approach. J. Environ. Manag..

[42] Hu J., Wang Z., Lian Y., Huang Q. (2018). Environmental regulation, foreign direct investment and green technological progress: Evidence from Chinese manufacturing industries. Int. J. Environ. Res. Public Health.

[43] Huang J.H., Yang X.G., Cheng G., Wang S. (2014). A comprehensive eco-efficiency model and dynamics of regional eco-efficiency in China. J. Clean. Prod..

[44] Yang L., Zhang X. (2016). Assessing regional eco-efficiency from the perspective of resource, environmental and economic performance in China: A bootstrapping approach in global data envelopment analysis. J. Clean. Prod..

[45] Yu Y., Chen D., Zhu B. (2013). Eco- efficiency trends in China, 1978–2010: Decoupling environmental pressure from economic growth. Ecol. Indic..

[46] Chu J.F., Zhu Q.Y., An Q.X., Xiong B. (2016). Analysis of China’s regional eco-efficiency: A DEA two-stage network approach with equitable efficiency decomposition. Comput. Econ..

[47] Feng Z., Zeng B., Ming Q. (2018). Environmental regulation, two-way foreign direct investment, and green innovation efficiency in China’s manufacturing industry. Int. J. Environ. Res. Public Health.

[48] Wang J.R., Zhang Y. (2018). Environmental regulation, green technological innovative intention and green technological innovative behavior. Stud. Sci. Sci..

[49] Baron R.M., Kenny D.A. (1986). The moderator-mediator variable distinction in social psychological research: Conceptual, strategic, and statistical considerations. J. Personal. Soc. Psychol..

